# LMS-based continuous reference values for the myokine musclin in serum in children and adults

**DOI:** 10.3389/fendo.2026.1822121

**Published:** 2026-05-08

**Authors:** Anne Schön, Hannah Weber, Katharina Schermuly, Anna Tschirner, Malgorzata Szaroszyk, Thomas Rebe, Jens Drube, Nele Kanzelmeyer, Ulrich Baumann, Anibh M. Das, Dieter Haffner, Maren Leifheit-Nestler

**Affiliations:** 1 Department of Pediatric Kidney, Liver, Metabolic and Neurological Diseases, Hannover Medical School, Hannover, Germany; 2 Department of Occupational Medicine, Hannover Medical School, Hannover, Germany; 3 Department of Pediatric Pneumology, Allergy and Neonatology, Hannover Medical School, Hannover, Germany

**Keywords:** adults, children, musclin, osteocrine, percentiles, reference values

## Abstract

**Background:**

Musclin is a myokine which is linked to muscle activity and systemic metabolic regulation. Age-specific reference values for serum musclin are lacking in both the pediatric and adult population, limiting the interpretation of circulating musclin concentrations in clinical practice and studies.

**Objectives:**

To establish Lambda–Mu–Sigma (LMS)-based continuous age-specific reference values for serum musclin in children and adults.

**Methods:**

Musclin concentrations were measured in serum by enzyme-linked immunosorbent assay (ELISA) in 399 children (190 girls) aged 0.1–18 years from the HAnnover Reference values for Pediatrics (HARP) cohort and in 502 adults (257 female) aged 18–70 years from the HAnnover Reference values for Adults (HARA) cohort. LMS-based continuous reference percentiles were generated using RefCurv software.

**Results:**

Serum musclin concentrations were significantly associated with age (p<0.001) but not with sex. Across the 3rd–75th percentiles, musclin concentrations increased continuously from infancy to adulthood. In the 75th–97th percentiles, serum musclin rose up to approximately 40–55 years of age and then showed a gradual decline until 70 years. Women taking estrogen-containing oral contraceptives had significantly lower musclin serum concentrations compared to age-matched women not using hormonal contraception.

**Conclusions:**

This study provides the first LMS-based continuous pediatric and adult reference percentiles for serum musclin, enabling the calculation of age-adjusted patient z-scores. This facilitates test result interpretation in children and adults in the clinic and studies.

## 1 Introduction

Skeletal muscle accounts for around 40-45% of total body mass and was long regarded mainly as an organ responsible for force generation and movement (1). Over the past two decades, however, it has become clear that muscle tissue is also a hormonally responsive and secretory organ that influences numerous bodily functions via myokines, e.g. interleukine-6, irisin, myostatin, brain-derived neutrotrophic factor, myonectin and meteorin-like (1–8) Myokines act in autocrine, paracrine, and endocrine fashions and regulate metabolism, the cardiovascular system, immune responses, and inter-organ communication (1, 4–10). One particularly prominent myokine candidate is musclin, also known as osterocrin (OSTN) (11, 12). Musclin is expressed predominantly in skeletal muscle and, to a lesser extent, in bone, and links muscle activity to systemic metabolic and cardiovascular regulation (13). Its secretion is stimulated by mechanical loading and exercise. Musclin binds to the natriuretic peptide clearance receptor NPR-C, thereby inhibiting the breakdown of natriuretic peptides (NPs) and enhancing cGMP-dependent signaling. This axis is important for the kidney, where NPs control renal hemodynamics, sodium excretion, and filtration (14, 15). In animal models of nephrotic and ischemic kidney injury, musclin protects against podocyte and tubular damage, reduces albuminuria, inflammation, and fibrosis, and improves glomerular structure (15–18). Systemic hemodynamic effects have also been described in sheep models showing that exogenous musclin increases circulating NPs and renal cGMP production while lowering blood pressure and central venous pressure, without eliciting marked natriuresis (19). Musclin thus appears to be an important mediator of muscle-kidney communication, and a potential renoprotective factor under systemic stress.

Beyond the kidney, musclin exhibits pleiotropic actions. Metabolically, elevated musclin concentration affects energy balance by suppressing thermogenesis in beige adipose tissue via Tfr1, thereby aggravating diet-induced obesity and metabolic disturbances (20). In the cardiovascular system, musclin acts cardioprotective, e.g. exercise-induced musclin improves mitochondrial function (21), protects the myocardium during ischemia-reperfusion, and attenuates hypertrophic remodeling under pressure overload, while lack of musclin worsens cardiac outcomes (22). In human umbilical vein endothelial cells musclin treatment ameliorated the expression of inflammation markers (phosphorylated NFκB and IκB) (23) and exerts anti-inflammatory effects via the PPARα/HO-1 pathway, thereby mitigating the interaction between endothelial cells and monocytes (23).

Additional data suggest contributions to tumor biology (anti-cachectic effects, metabolic stress) (24), regulation of fibro-adipogenic progenitor cells in muscle (25), pulmonary vascular homeostasis (26) and the muscle-brain axis, where it exerts antidepressant effects via hypothalamic urocortin-2 signaling (27).

In a recent study of young inactive men, musclin levels rose with exercise intensity and were positively correlated with those of ANP. Additionally, a potential interplay between NPR-C expression and musclin dynamics on ANP was suggested (28). Taken together in humans, musclin concentration rise after exercise, in obesity, insulin resistance, and type 2 diabetes (20, 29–31), whereas low concentrations are associated with unfavorable outcomes in cardiovascular diseases, for example after TAVI and in patients with aortic stenosis (22, 32). Musclin thus appears to be a sensitive but nonspecific marker of metabolic strain, muscle status, and systemic stress.

The musculoskeletal system undergoes fundamental changes as the body matures. It evolves from a highly adaptable, growth-oriented state in childhood to a phase of maximum structural stability in young adulthood (33–35). This transition is primarily driven by hormonal influences and mechanical stress. Typical characteristics include an increase in muscle fiber size, increasing tendon stiffness, and the formation of stable, highly mineralized bones (36). This developmental phase is crucial for building high bone and muscle mass, which significantly determines how quickly these tissues are broken down later in life and how high the risk is for musculoskeletal disorders such as osteosarcopenia (37). It is evident that bone mass increases continuously during childhood and rises particularly sharply during puberty, with an increase in bone mineral content of approximately 35–50%. The peak is usually reached between the ages of 20 and 30 (38, 39). However, during periods of intense growth, bones are often not yet fully mineralized, which increases the risk of fractures in adolescents (40). At the same time, muscle mass and muscle strength, particularly in the legs, increase linearly with age in children. Adolescents experience high rates of muscle mass acquisition and strength. Muscle mass and strength generally peak around 26 to 35 years old. After the age 30, muscle performance begins to decline, with a more rapid decline in strength and muscle mass (sarcopenia) occurring after 65–70 years of age, increasing the risk of disability (41) In addition, there are marked changes in the rate of muscle relaxation during childhood: it doubles between the ages of 3 and 10 and reaches nearly adult levels by around age 10. As children grow older, their tendons also become stiffer and can transmit forces more efficiently; tendon stiffness comparable to that of adults is usually achieved around the time of peak physical growth around age 14 (34, 42).

The development of the musculoskeletal system can influence the production and regulation of the myokine musclin, which is released by bones and muscles. So far, the interpretation of serum musclin concentrations in clinical practice and studies is limited by the lack of standardized assays and reference values. Available ELISA tests are restricted to research use and differ markedly in sensitivity and calibration. Previous published cut-off values therefore cannot be considered true reference intervals. Normal ranges for serum musclin from children and adults have not been established. Given the dynamic age-related changes in the musculoskeletal system during maturation and aging mentioned above, it is particularly important to establish age- and sex-specific reference values for musclin. We hypothesized that serum musclin concentration depends on age and/or sex. The Lambda–Mu–Sigma (LMS) method allows to generate continuous reference percentiles for parameters irrespectively on the presence of a normal distribution, which is usually lacking when assessing parameters across ages, especially when including children (43–46).

Here we provide continuous age-dependent LMS reference percentiles for serum musclin in children and adults, thereby creating a basis for future clinical and translational research based on age-related z-scores of serum musclin.

## 2 Methods

### 2.1 Participants and study design: pediatric and adult cohorts

The HAnnover Reference Values studies comprise two single-center, cross-sectional investigations designed to establish sex-specific reference ranges for key laboratory biomarkers across the lifespan. They were performed in compliance with the Declaration of Helsinki and was approved by the Ethics Committee of the Hannover Medical School (#11127_BO_S_2023). All participants or their caregiver (for children <16 years of age) provided written informed consent prior to enrollment.

The HAnnover Reference Values for Pediatrics (HARP) study was initiated in 2021 and included children aged 0.1 to 18.0 years recruited from the outpatient services of Hannover Medical School. The HAnnover Reference Values for Adults (HARA) study was launched in 2024 as a prospective cross-sectional study and enrolled individuals aged 18 to 70 years, primarily from occupational health examinations and preventive medical assessments at Hannover Medical School and affiliated outpatient clinics, as well as volunteers from the surrounding community. Both studies applied strict exclusion criteria to ensure inclusion of healthy participants without conditions potentially affecting growth, nutritional status, skeletal or muscular integrity, or systemic inflammation. Exclusion criteria comprised growth disorders, malnutrition, diabetes mellitus, reduced mobility, muscle weakness, recent fractures, bone or muscular diseases, inflammatory or hepatic disorders, anemia, impaired renal function or proteinuria, defined as a urinary protein-to-creatinine ratio exceeding 0.3 g/g (47) or a reduced age-related eGFR (48), or medication potentially impairing muscle, bone and mineral metabolism. Participants with evidence of infection, indicated by elevated C-reactive protein concentration, were also excluded. Additional adult-specific exclusion criteria included pregnancy and a history of malignancy within the past 10 years.

**Figure 1:**
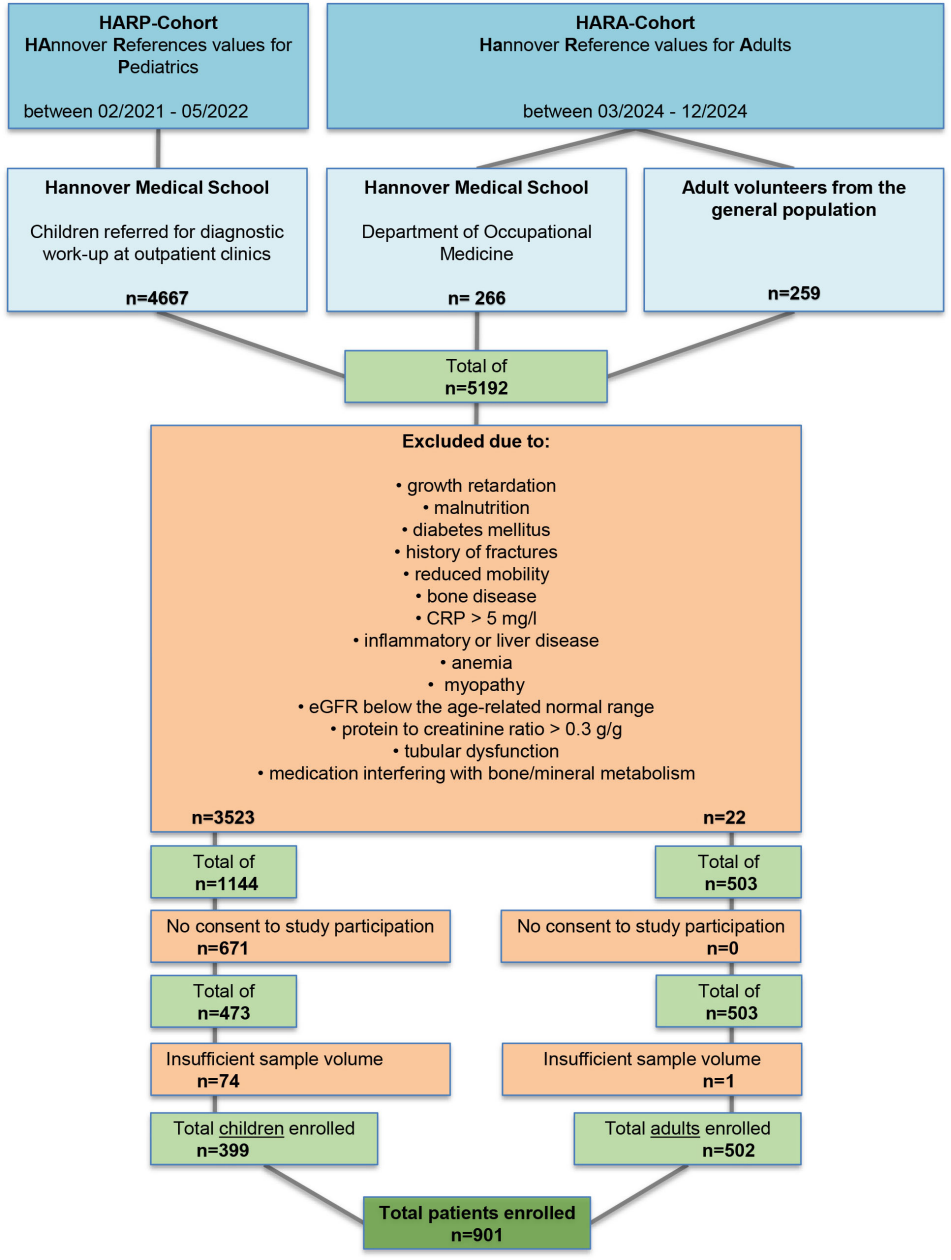
Flow chart for the selection of participants in the HAnnover Reference values for Pediatrics (HARP) study and the HAnnover Reference values for Adults (HARA) study for the measurement of musclin. A total of 4667 children and 525 adults were recruited from the Hannover Medical School (MHH) and the medical office of the MHH and volunteers from the general population, respectively. After strong exclusion criteria and based on the availability of informed consent forms and biological samples, 399 children and 502 adults were included in the study, respectively. CRP, C-reactive protein; eGFR, estimated glomerular filtration rate.

The minimum sample size was calculated in advance. The goal was to include at least 5 samples per age group and sex for the children and at least 3 samples per age group and sex for the adults. For the present analysis, 399 children (47.6% female; median age 11 years [IQR 5–14.5]) and 502 adults (51.2% female; median age 36.5 years [IQR 27.2–54.1]) were included after screening (**Figure 1**; **Table 1**).

**Table 1:** Demographic, anthropometric and biochemical parameters of the HARA and HARP cohort.

Variables	All (n= 901)	Adults (n=502)	Children (n=399)	P value
Sex, female n/n (%)	356/703 (50.6)	257/502 (51.2)	190/399 (47.6)	0.286
Age, years	23.7 (12.2; 40.8)	36.5 (27.2; 54.1)	11.1 (5.1; 14.5)	< 0.001
Height, cm	168 (154, 178)	175 (168, 182)	147 (110, 165)	< 0.001
Height, z-score	0.2 (-0.5; 1.0)	0.4 (-0.3; 1.0)	-0.1 (-0.7; 0.9)	< 0.001
Body weight, kg	65.0 (47.1; 80.0)	75.0 (63.8; 86.1)	39.9 (18.8; 59.7)	< 0.001
Body weight, z-score	0.5 (-0.2; 1.2)	0.7 (-0.1; 1.3)	0.2 (-0.7; 1.0)	< 0.001
BMI, kg/m^2^	22.0 (18.0; 25.0)	24.1 (21.0; 26.1)	18.0 (15.1; 22.1)	< 0.001
BMI, z-score	0.26 (-0.49; 0.95)	0.48 (-0.35; 1.06)	0.00 (-0.94; 0.85)	< 0.001
CRP, mg/L	0.7 (0.6; 1.4)	0.8 (0.6; 1.5)	0.6 (0.6; 0.97)	< 0.001
Creatine kinase, U/L	107.5 (80.0; 156.0)	105.1 (78.2; 155.3)	125.8 (91.0; 164.0)	0.068
Creatinine, µmol/L	69.0 (50.1; 83.2)	79.1 (70.1; 90.2)	46.5 (32.7; 59.0)	< 0.001
Cystatin C, mg/L	0.9 (0.8; 0.9)	0.8 (0.8; 0.9)	0.9 (0.8; 1.0)	0.091
eGFR, ml/min/1.73 m^2^	96.2 (82.4; 110.2)	87.8 (77.5; 99.9)	110.2 (95.1; 126.5)	< 0.001
UACR, mg/g creatinine	5.8 (3.7; 11.3)	5.4 (3.5; 11.3)	6.1 (3.9; 11.2)	0.151
Musclin, ng/ml	5.2 (3.2; 7.9)	6.1 (4.0; 8.9)	4.0 (2.3; 6.4)	< 0.001

Data are presented as median (IQR) for non-normally distributed data or as percentage (%), determined by the Shapiro-Wilk test; P values were calculated using Mann-Whitney-U test or Chi-squared test, respectively. BMI, body mass index; CRP, C-reactive protein; eGFR, estimated glomerular filtration rate, UACR, urinary albumin to creatinine ratio.

### 2.2 Investigations and biomarker analysis

Blood samples were collected between 8 a.m. and 12 a.m. from children and between 8 a.m and 2 p.m. from adults, irrespective of the fasting state. Plasma/serum samples were stored at -80 C until assayed. In addition to routine demographic and anthropometric assessments, adult participants were asked to complete a standardized questionnaire which collected information on smoking status, habitual physical activity (any kind of sport) per week, and — for female participants — the use of estrogen-containing oral contraceptives (OC) or estrogen-based oral therapies (OE) for menopausal symptom management.

Routine clinical chemistry analyses were performed for serum C-reactive protein (CRP), creatine kinase, creatinine, cystatin C, and urinary concentrations of calcium, phosphate, protein, albumin, glucose, and creatinine. All measurements were obtained using automated assays on the Cobas 8000 platform (module c701; Roche Diagnostics, Mannheim, Germany).

Estimated GFR was calculated using the creatinine-based equation developed by the European Kidney Function Consortium (EKFC), as described by Pottel et al. (49). ELISA kits were used to quantitatively determine the serum concentration of musclin (Biomatik, Catalog Number: EKC34895-96T, detection range 0.312–20 ng/ml, sensitivity 0.078 ng/ml, intra-assay precision CV <8%, inter-assay precision CV <10%). The assays were conducted in accordance with the manufacturer´s instructions. Each sample was measured in duplicate using the Tecan Infinite M200 Pro, with quantitative evaluation performed through Magellan 7.2 software.

### 2.3 Statistical analysis

Descriptive statistics are presented as median and interquartile range (IQR) for non-normally distributed data determined by the Shapiro–Wilk test, as mean with standard deviation (SD) for normally distributed data, or as percentages. Differences between groups were assessed using Mann–Whitney U-test for non-normally distributed continuous variables and the chi-squared test for categorical variables. Bivariate correlations were performed by Spearman correlation tests with a two-tailed test of significance. Statistical significance was defined as p <0.05. All analyses were performed using SPSS software, version 30.0 (IBM Corporation, New York, USA), and GraphPad Prism, version 10.4.2.

Age-specific percentile reference curves were generated using the LMS method in RefCurv 0.4.2 (Windows) (46) implemented via generalized additive models for location, scale, and shape (GAMLSS) as described by Rigby and Stasinopoulos (50). The LMS method characterizes the distribution at each age through three parameters: Lambda (L), representing skewness; Mu (M), representing the median; and Sigma (S), reflecting the coefficient of variation, according to the original formulation by Cole and Green (51).

## 2 Results

### 2.1 Baseline characteristics of the participants

A total of 901 participants (447 female) fulfilled the criteria for this reference study. The median age was 23.7 years (IQR 12.2; 40.8). The z-scores for height, weight, BMI and eGFR were within the normal range (**Table 1**). Serum creatinine kinase concentrations were within the normal range in all participants. In the HARA cohort about 15% were smoker. Estrogens, including OC and OE were taken by 26% percent of adult female participants (OC in 24% and OE in 2%). Physical activity in adult participants varied, with 17% of adults exercising for less than 30 minutes a week, 23% for 30–59 minutes, 26% for 60–120 minutes, and 32% for more than 120 minutes.

### 2.2 LMS percentiles for serum musclin

**Figure 2:**
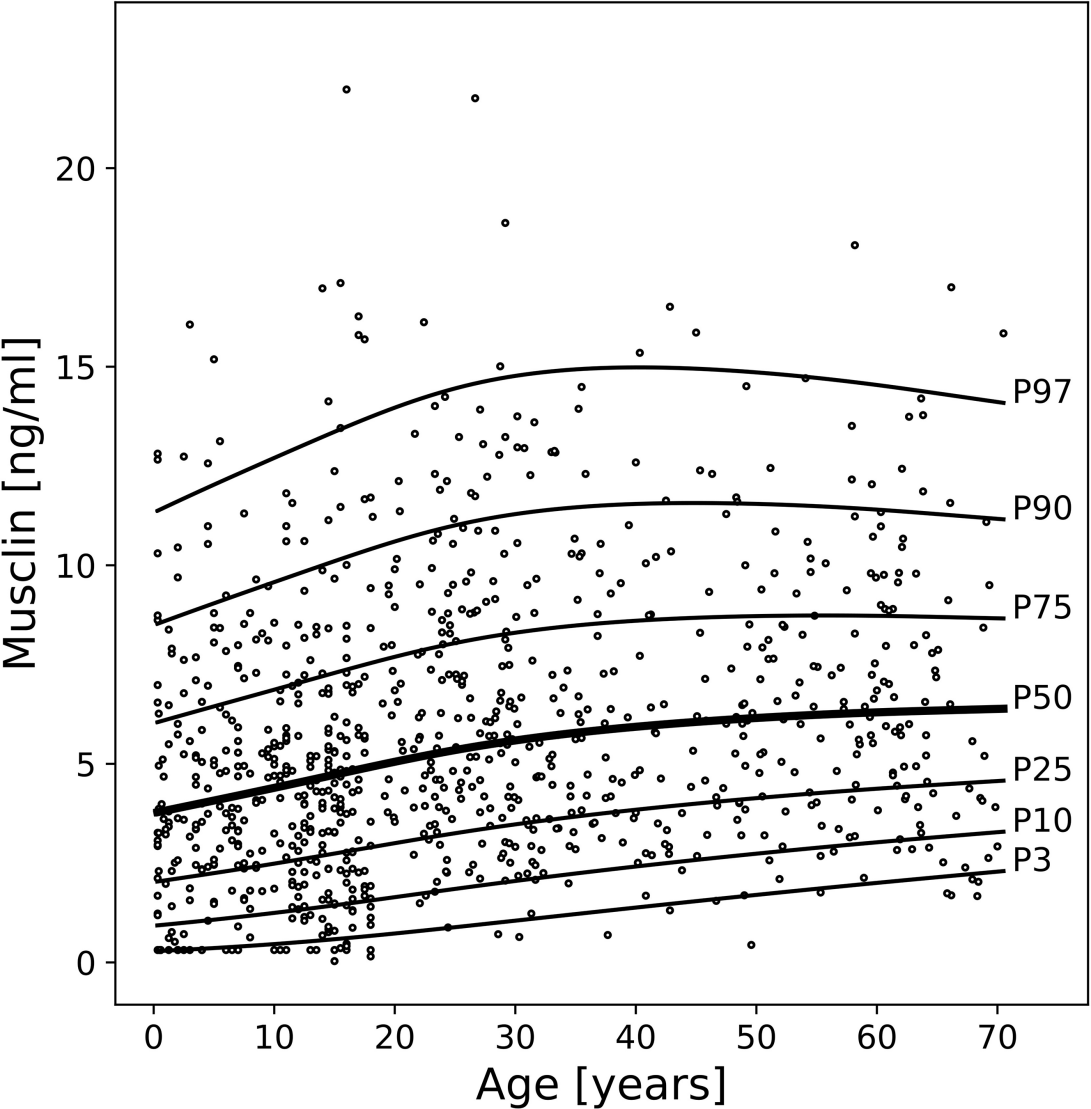
LMS percentiles for serum musclin according to age. The 3th, 10th, 25th, 50th (bold line), 75th, 90th, and 97th percentiles are given.

Serum musclin concentrations were significantly associated with age (p<0.0001), but not with sex (p=0.286). Therefore, age-specific percentile limits and LMS values for musclin for both sexes are provided (**Table 2**; **Figure 2**). Serum musclin concentrations continuously increased from infancy through adulthood, followed by a plateau in the fifth decade of life. In early childhood, median musclin concentration (P50) ranged from approximately 3.7 ng/ml at birth to about 4.1 ng/ml by school age. During adolescence, median concentrations reached approximately 4.9 ng/ml. In adulthood, musclin concentration continued to increase gradually, reaching around 6.0–6.4 ng/ml in older adults. Higher percentiles (P75, P90, P97) followed the same trajectory, indicating a consistent age-related rise across the entire distribution (**Table 2**).

**Table 2:** Age-specific percentile limits and LMS values for musclin (ng/ml) in children and adults.

Age	3rd	10th	25th	50th	75th	90th	97th	M	S	L
0	0.282	0.924	2.024	3.762	6.0223	8.492	11.327	3.73	0.799	0.52
1	0.296	0.953	2.068	3.824	6.1079	8.604	11.47	3.796	0.793	0.52
2	0.311	0.982	2.112	3.887	6.1932	8.715	11.612	3.861	0.787	0.52
3	0.327	1.013	2.157	3.95	6.2781	8.825	11.753	3.926	0.781	0.51
4	0.344	1.044	2.203	4.013	6.3627	8.934	11.892	3.991	0.776	0.51
5	0.361	1.076	2.249	4.076	6.4471	9.042	12.029	4.056	0.770	0.51
6	0.379	1.108	2.295	4.139	6.5311	9.15	12.166	4.121	0.764	0.50
7	0.399	1.142	2.342	4.203	6.6151	9.257	12.301	4.187	0.758	0.50
8	0.419	1.176	2.390	4.267	6.6991	9.363	12.436	4.252	0.753	0.50
9	0.440	1.211	2.438	4.331	6.7832	9.470	12.57	4.318	0.747	0.49
10	0.461	1.247	2.487	4.396	6.8674	9.576	12.703	4.384	0.741	0.49
11	0.484	1.283	2.537	4.461	6.9517	9.682	12.836	4.451	0.736	0.49
12	0.508	1.321	2.587	4.527	7.0362	9.788	12.969	4.518	0.730	0.48
13	0.532	1.359	2.638	4.593	7.1207	9.894	13.100	4.585	0.725	0.48
14	0.558	1.398	2.689	4.659	7.2052	9.999	13.231	4.652	0.720	0.48
15	0.584	1.437	2.741	4.726	7.2895	10.100	13.361	4.719	0.714	0.47
16	0.611	1.478	2.793	4.792	7.3732	10.210	13.489	4.787	0.709	0.47
17	0.64	1.518	2.846	4.858	7.4559	10.31	13.614	4.853	0.704	0.47
18	0.668	1.559	2.898	4.924	7.5374	10.41	13.736	4.92	0.698	0.46
19	0.698	1.601	2.95	4.989	7.6172	10.51	13.855	4.985	0.693	0.46
20	0.728	1.643	3.002	5.052	7.6949	10.6	13.969	5.049	0.688	0.46
21	0.759	1.684	3.054	5.115	7.7701	10.69	14.078	5.112	0.683	0.45
22	0.79	1.726	3.105	5.175	7.8427	10.78	14.181	5.173	0.678	0.45
23	0.822	1.768	3.155	5.235	7.9123	10.86	14.278	5.233	0.673	0.44
24	0.854	1.81	3.204	5.292	7.9786	10.93	14.369	5.29	0.668	0.44
25	0.886	1.851	3.252	5.347	8.0415	11.01	14.453	5.346	0.663	0.44
26	0.919	1.892	3.299	5.400	8.1009	11.07	14.530	5.399	0.658	0.43
27	0.952	1.932	3.345	5.451	8.1566	11.13	14.600	5.450	0.653	0.43
28	0.985	1.972	3.39	5.499	8.2086	11.19	14.663	5.498	0.648	0.43
29	1.018	2.012	3.434	5.545	8.257	11.24	14.719	5.545	0.643	0.42
30	1.051	2.051	3.476	5.589	8.3021	11.29	14.769	5.589	0.638	0.42
31	1.084	2.089	3.517	5.631	8.3440	11.33	14.812	5.631	0.634	0.42
32	1.117	2.127	3.557	5.671	8.3829	11.37	14.850	5.671	0.629	0.41
33	1.150	2.165	3.596	5.710	8.4189	11.40	14.883	5.710	0.624	0.41
34	1.184	2.202	3.635	5.746	8.4523	11.43	14.910	5.746	0.620	0.41
35	1.217	2.239	3.672	5.781	8.4831	11.46	14.933	5.781	0.615	0.40
36	1.250	2.275	3.708	5.815	8.5115	11.48	14.951	5.814	0.610	0.40
37	1.283	2.311	3.743	5.846	8.5375	11.50	14.965	5.846	0.606	0.40
38	1.315	2.346	3.778	5.877	8.5614	11.52	14.974	5.877	0.601	0.39
39	1.348	2.381	3.811	5.906	8.5832	11.53	14.980	5.906	0.597	0.39
40	1.381	2.415	3.844	5.934	8.6032	11.54	14.983	5.934	0.592	0.39
41	1.413	2.449	3.876	5.960	8.6214	11.55	14.982	5.960	0.588	0.38
42	1.446	2.483	3.908	5.986	8.6379	11.56	14.978	5.986	0.584	0.38
43	1.478	2.516	3.938	6.010	8.6528	11.56	14.971	6.010	0.579	0.38
44	1.510	2.549	3.969	6.033	8.6663	11.57	14.962	6.033	0.575	0.37
45	1.542	2.581	3.998	6.056	8.6783	11.57	14.950	6.056	0.571	0.37
46	1.574	2.613	4.027	6.077	8.689	11.57	14.936	6.077	0.566	0.37
47	1.606	2.645	4.055	6.098	8.6985	11.56	14.919	6.098	0.562	0.36
48	1.637	2.676	4.083	6.118	8.7068	11.56	14.900	6.118	0.558	0.36
49	1.669	2.707	4.110	6.136	8.7139	11.55	14.880	6.136	0.554	0.36
50	1.700	2.738	4.136	6.155	8.720	11.55	14.857	6.155	0.55	0.35
51	1.731	2.768	4.162	6.172	8.7251	11.54	14.833	6.172	0.546	0.35
52	1.762	2.798	4.188	6.189	8.7291	11.53	14.807	6.189	0.542	0.35
53	1.793	2.828	4.213	6.205	8.7322	11.52	14.779	6.205	0.537	0.34
54	1.824	2.857	4.237	6.220	8.7342	11.50	14.749	6.220	0.533	0.34
55	1.854	2.886	4.261	6.234	8.7354	11.49	14.718	6.234	0.530	0.34
56	1.884	2.914	4.285	6.248	8.7355	11.47	14.686	6.248	0.526	0.33
57	1.914	2.942	4.308	6.261	8.7348	11.46	14.651	6.261	0.522	0.33
58	1.944	2.970	4.330	6.274	8.7332	11.44	14.616	6.274	0.518	0.33
59	1.973	2.998	4.352	6.286	8.7306	11.42	14.579	6.286	0.514	0.32
60	2.003	3.025	4.373	6.297	8.7272	11.40	14.540	6.297	0.510	0.32
61	2.032	3.051	4.394	6.307	8.7229	11.38	14.500	6.307	0.506	0.32
62	2.060	3.077	4.415	6.317	8.7178	11.36	14.459	6.317	0.503	0.31
63	2.089	3.103	4.434	6.326	8.7121	11.34	14.417	6.326	0.499	0.31
64	2.117	3.129	4.454	6.335	8.7058	11.31	14.374	6.335	0.495	0.31
65	2.146	3.154	4.473	6.343	8.6992	11.29	14.330	6.343	0.491	0.30
66	2.174	3.179	4.492	6.351	8.6923	11.26	14.286	6.351	0.488	0.30
67	2.202	3.204	4.511	6.359	8.6852	11.24	14.242	6.359	0.484	0.30
68	2.230	3.229	4.530	6.367	8.6782	11.22	14.198	6.367	0.480	0.29
69	2.257	3.254	4.549	6.375	8.6713	11.19	14.155	6.375	0.477	0.29
70	2.285	3.279	4.567	6.383	8.6644	11.17	14.111	6.383	0.473	0.29
71	2.313	3.304	4.586	6.391	8.6577	11.15	14.068	6.391	0.470	0.28

To calculate age-related z-scores for serum muslin, the determined concentration x together with the corresponding age-related values for L, M, and S are used according to the formula:.

z-score = ((x/M)^L^-1)/(S×L). (für L ≠ 0) L, skewness; M, median; S, coefficient of variation.

### 2.3 Serum musclin correlates with oral contraceptive use in women but not with physical activity or smoking in adults

**Figure 3:**
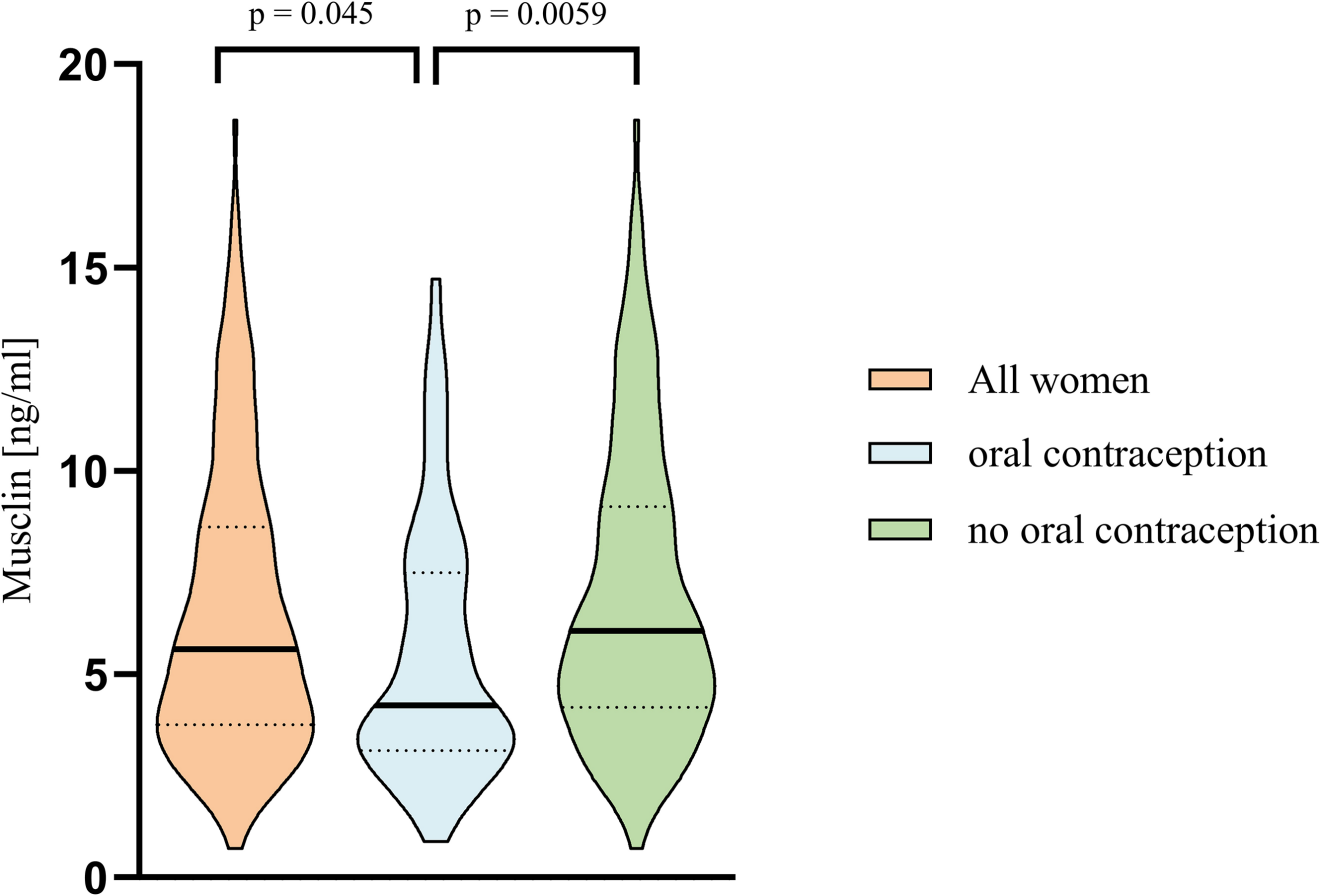
Violin plot showing the distribution of serum musclin in all women aged 18 to 54 years (left), women on estrogen containing oral contraceptives (middle) and those who do not take oral conception (right). The p-value was calculated using Mann-Whitney-U test.

In women aged 18–54 years, serum median musclin concentration was significantly lower in those on OC (4.23 ng/ml [IQR 3.12; 7.49]) compared to their peers not taking OC (5.62 ng/ml IQR 3.76; 8.62], p = 0.045) (**Figure 3**).

**Figure 4:**
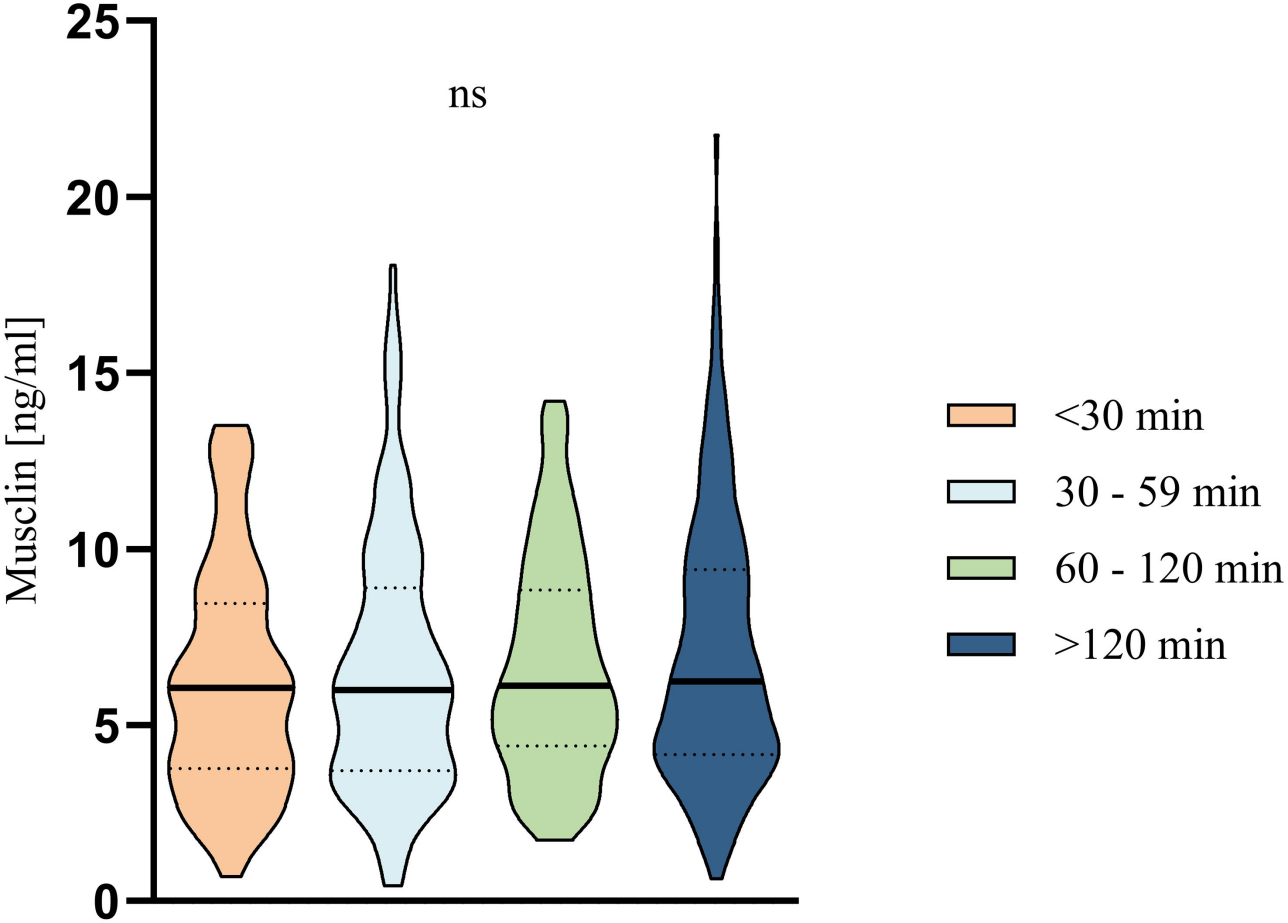
Violin plot showing the distribution of serum musclin in adult participants according to weekly physical activity in minutes (min); <30 min, 30–59 min, 60–120 min, <120 min. Statistics were performed using Mann-Whitney U test. ns, not significant.

Serum musclin concentrations did not significantly differ between predefined physical activity groups in adults (**Figure 4**) or between smokers and non-smokers (6.5 ng/ml [IQR 3.8;9.2] versus 6.1 ng/ml [IQR 4.2-8.8], p=0.809).

## 3 Discussion

To our knowledge, this is the first study to establish continuous, age-specific LMS-based reference values for circulating musclin across all age groups. The availability of developmental percentile curves provides a crucial foundation for interpreting serum musclin concentrations in both clinical and research settings, particularly because musclin has recently attracted attention as a biomarker linking skeletal muscle biology, NP signaling, and systemic organ function (15, 20, 27).

From the nephrological perspective, the establishment of normative musclin values is of particular importance. Musclin is a known modulator of the natriuretic peptide (NP) system through binding to the clearance receptor NPR-C, enhancing NP bioavailability and downstream cGMP signaling (15). Given the central role of NP in renal hemodynamics, sodium handling, and glomerular filtration, alterations in musclin signaling may have direct implications on kidney function and disease progression. Experimental evidence strongly supports a renoprotective role of musclin. In murine models of adriamycin-induced nephropathy, musclin overexpression ameliorated podocyte injury, reduced albuminuria, and preserved glomerular structure, effects mediated in part via inhibition of p38 MAPK signaling and enhancement of GC-A–cGMP pathways (17).

Previous clinical studies have reported associations between serum musclin concentration based on absolute values and adverse outcomes in adults, but none provided age-adjusted normative data. In patients undergoing transcatheter aortic valve implantation (TAVI), low circulating musclin concentrations were identified as an independent predictor of 1-year mortality, even after adjustment for established clinical risk scores. Individuals with low serum musclin also presented with a greater burden of comorbid conditions, including arterial hypertension, prior stroke, frailty, low albumin, and elevated NT-proBNP (32). Importantly, none of these conditions were present in our reference population, as individuals with known cardiovascular disease were excluded. Consequently, the low serum musclin concentrations associated with adverse prognosis in TAVI patients would probably not have been observed when using our reference values, underscoring the fundamental distinction between disease-focused cohorts and population-based reference datasets.

Beyond cardiovascular disease, several studies have suggested a potential link between musclin and metabolic health (22, 25, 52). Low musclin concentration has been reported in association with obesity or impaired metabolic regulation, whereas experimental data in male mice demonstrate that musclin can suppress beige fat thermogenesis and impair systemic energy homeostasis via Tfr1–PKA signaling (20). Although these mechanistic insights cannot be directly extrapolated to humans, they support the concept that musclin is not merely a passive circulating marker but may interact with pathways relevant to whole-body metabolic balance.

Taken together with findings from TAVI cohorts, these data highlight that musclin can deviate from normative ranges in both directions—towards unusually low or high concentrations—depending on the underlying pathophysiological state. This bidirectional variability underscores the need for reliable reference values to distinguish normal, age-related patterns from disease-associated alterations.

Moreover, emerging experimental literature further reinforces the biological significance of musclin. Recent animal studies demonstrate that musclin is a key determinant of the fibro-adipogenic progenitor (FAP) and thereby plays a central role in muscle tissue homeostasis and regeneration. Exercise-induced increases in musclin limit fibrosis and fatty infiltration following muscle injury or disuse by suppressing FAP proliferation, promoting apoptosis via Filamin A interacting protein 1 like (FILIP1L), and facilitating macrophage-mediated clearance of apoptotic cells. The loss of this musclin–FILIP1L axis abolishes the beneficial effects of exercise on muscle repair, highlighting musclin as an active regulator of tissue remodeling rather than a passive marker (25). These mechanistic insights, together with observations in metabolic and cardiovascular models, suggest that musclin participates in diverse physiological and pathophysiological pathways relevant to systemic health.

A further challenge in establishing musclin as a clinically interpretable biomarker is the lack of standardized diagnostic testing. Currently, all musclin assays are intended exclusively for research use, and available ELISAs differ markedly in analytical sensitivity, calibration ranges, and overall performance characteristics. In the present literature, the frequently cited “first” study on human musclin did not employ an ELISA but rather a commercial radioimmunoassay (RIA) from the Beijing Sino-UK Institute of Biological Technology (curve range 0 to 400 ng/L, sensitivity 1,25 pg/ml) (53), which limits direct comparability with later work using ELISA-based methods.

Subsequent clinical and translational studies assessing circulating musclin have relied on sandwich ELISAs for plasma, serum and tissue homogenates. The following sandwich ELISAs have been used: Biomatik (Catalog Number: EKC34895-96T, detection Range 0.312–20 ng/ml, sensitivity 0.078 ng/ml, intra-assay precision CV <8%, inter-assay precision CV <10%) described by Szaroszyk and colleagues (20), a human osteocrin/musclin ELISA from CUSABIO (Catalog Number: CSB-E12021h, detection range 0.312–20 ng/ml, sensitivity 0.078 ng/ml) (32) and a sandwich ELISA by LifeSpan (LS-F7799, detection range 15.6–1000 pg/ml, sensitivity 6.1 pg/ml) used by Sanchez et al. and Clark et al. (30).

Since our working group has extensive experience with the Biomatik musclin ELISA, and the detection range and sensitivity are comparable to those of the Cusabio assay, we chose Biomatik for establishing and reporting normal serum musclin values. Two previous studies using a LifeSpan ELISA reported markedly divergent concentrations in healthy controls, making it difficult to derive reliable reference values from that data (30, 54). Moreover, one of the studies reported the results in ng/ml even though the ELISA readout is in pg/ml, further questioning the comparability and validity of those reported concentrations.

Consequently, published studies have relied on assay-specific thresholds rather than true population-based reference intervals. A recently published study in humans showed an increase in serum musclin concentrations following aerobic exercise (28). The later exchange on this publication between Prickett and Espiner (55) and the response by Y. Kim (56) highlighted important methodological concerns regarding the measurement of musclin, particularly the specificity and reliability of ELISA assays.

Within this framework, our LMS-based reference values provide an essential baseline for clinical and scientific interpretation. By defining the normal developmental trajectory of serum musclin from infancy through older adulthood, our data enable future studies to investigate whether deviations from these percentiles—whether lower levels, as described in TAVI patients, or markedly elevated levels in acute catabolic stress states—reflect altered musclin biology with clinical relevance. These normative values may be particularly valuable in pediatric and adult studies in which serum musclin has not yet been systematically explored. Additionally future research can more rigorously examine associations between serum musclin and metabolic markers, inflammatory profiles, NPs, indices of muscle mass or function, and other systemic biomarkers. Such studies will be instrumental in clarifying whether serum musclin serves primarily as a correlate of muscle physiology or may act as a broader biomarker, reflecting systemic health and disease susceptibility.

Our study has limitations. Most of the study population was of Caucasian descent. Therefore, the results should not be applied to different ethnicities without further research. In addition, sampling was not conducted strictly in the fasting state, which may have biased the results. Finally, we did not account for potential circadian variations of serum musclin concentration.

Overall, these age-specific reference percentiles and LMS values enable the calculation of standardized z-scores to facilitate test result interpretation in children and adults. This lays the groundwork for future longitudinal and disease-focused investigations examining serum musclin as a biomarker of muscle–kidney crosstalk, systemic metabolic stress, and cardiovascular risk. Integration of these normative data with mechanistic and clinical studies may help clarify the role of musclin in health and disease and support its evaluation as a potential biomarker or therapeutic target.

## Data Availability

The original contributions presented in the study are included in the article/supplementary material. Further inquiries can be directed to the corresponding author.
